# Prevalence, Risk Factors, and Public Awareness of Internal Helminthes in Commercial Fish of Lake Chamo, Southern Ethiopia

**DOI:** 10.1155/japr/8839195

**Published:** 2026-03-04

**Authors:** Tagash Girma, Wasihun Seyoum, Tamirat Kaba, Ephrem Tora

**Affiliations:** ^1^ Department of Animal Science, Arba Minch University, Arba Minch, Ethiopia, amu.edu.et

**Keywords:** fish, internal helminthes, Lake Chamo, prevalence, public awareness, risk factor

## Abstract

Fish is a vital source of food, employment, trade, and economic well‐being for people. But, it is hindered by a variety of diseases caused by bacteria, viruses, fungi, and most commonly parasites. The impact and public health importance of parasitic helminthes in fish which cause devaluation, retarded growth, morbidity, and mortality have not been sufficiently studied in Ethiopia, especially in Lake Chamo. Therefore, this cross‐sectional study was conducted in Lake Chamo, Southern Ethiopia from October 2023 to December 2024 to estimate the prevalence, identify determinants, and assess community awareness towards public health importance of parasitic helminthes in commercially viable fish species. The study was performed using 369 commercially viable fish species selected from local fishermen either randomly or conveniently. A total of 156 respondents at vicinity of Lake Chamo were surveyed using structured questionnaire to assess their awareness level towards fish parasites. The overall prevalence of fish helminthic parasite infection was 67.8% (250/369) with high prevalence observed in *Oreochromis niloticus* (80.6%) and no parasite identified in *Hydrocynus vittatus*. Univariable logistic regression analysis showed that the odds of infection were statistically significant with more likely in *O. niloticus* (OR = 2.9, CI = 1.32–6.54), female (OR = 1.6, CI = 1.03–2.49), and in large standard length (OR = 2.2, CI = 1.15–4.16) compared with their respective categories. From identified fish helminthes, *Clinostomum* (57.67%) was dominant genus followed by cestode larvae (50.8%), *Contracaecum* (12.8%), Acanthocephala (6%), cestode (3.2%), and unidentified parasite (3.2%). The survey revealed widespread consumption of raw fish (90.4%) around Lake Chamo, coupled with a total absence (100%) of practical preventive measures among respondents. Generally, commercially viable fish in Lake Chamo are widely affected by internal helminthes, creating a risk of fish borne zoonosis due to poor management and low awareness level. Thus, integrated parasitic control approaches involving fish, humans, and the environment should be applied in the study area.

## 1. Introduction

Fish is a main source of animal protein for 20% of the world′s population, and as the human population is growing, increasing the price of meat and high preference for fish protein, there is a high demand for fish worldwide [1–3]. In consequence, attempts are now being made to enhance fish production systems through culture and capture fisheries [4]. In many developing countries including Ethiopia, the fishery sector is a crucial resource offering significant socioeconomic benefits. Its contributions to national economies are diverse, encompassing the improvement of food and nutrition security, the generation of livelihoods and employment for local communities, and the direct increase of gross domestic product (GDP) and foreign exchange earnings [3, 5].

Ethiopia has been a landlocked nation and its only source of fish is inland waterways. The country has a surface area estimated at 7334 km^2^ of major lakes and reservoirs and 275 km^2^ of small water bodies with 7185 km of rivers within the country. Some of these bodies of water are vital fishing grounds for the nation [6, 7]. In Ethiopia, the primary fish production system is mainly focused on catching fish from natural water bodies, such as lakes and rivers with very limited recent developments in fish farming [3, 5]. Also, there are about 168–183 different fish species found in the country and 37–57 of them are native to the nation [8, 9]. The most common and commercialized fish species in Ethiopia are Nile tilapia (*Oreochromis niloticus)*, African catfish *(Clarias gariepinus)*, Nile perch (*Lates niloticus*), *Bagrus* species *(Bagrus docmak*), and *Barbus* species [6, 10–12]. Despite a global annual consumption exceeding 20 kg per capita, Ethiopia currently records a much lower rate of 0.5 kg; nevertheless, the consumption is steadily increasing as the country taps into its estimated annual potential of 45,000–51,500 tons primarily supplied by six major lakes namely Tana, Ziway, Langano, Hawassa, Abaya, and Chamo [1, 5, 7].

Lake Chamo is one of the major lakes in rift valley, which found in the Southern Regional state of Ethiopia. With over 20 different species, the fish fauna of Lake Chamo is the most diverse of the rift valley lakes, second only to Lake Abaya [3, 7, 13]. The fishery of Lake Chamo has supported the livelihoods of the surrounding communities through fishing for decades. It also represents the rift valleys most productive fishery, primarily yielding *O. niloticus* and *L. niloticus* for key local and urban markets, including Addis Ababa [14].

Despite the availability of huge potential for fish production, Ethiopian fish production is affected by several natural and human‐made factors such as poor management systems, improper fishing methods like overexploitation, wrong place and time of fishing, presence of water hyacinth and pollution, traditional fish production techniques, introduction of exotic fish, presence of limited trained human resources, and inadequate legal and policy frameworks [6, 15, 16]. Despite the rising demand for fish, people do not pay as much attention to fish health issues as they do to those of other livestock animals [11]. Similar to livestock, fish are vulnerable to a variety of diseases brought on by environmental factors, management problems, and disease‐causing agents such as viruses, bacteria, fungi, and most importantly, parasites [17–19].

Different parasites can use freshwater fish as definitive, intermediate, or paratenic hosts at different stages of their life cycles [1, 18]. Parasitic diseases of fish are very common all over the world and are of particular importance in the tropics [15, 19]. Among the diverse array of internal and external fish parasites, internal helminthes are one of the most prevalent pathogen groups impacting fish health [11]. According to Shamsi [20] over 40 parasite species associated with seafood have been identified globally. These organisms are taxonomically classified into several major groups, including protozoa, cnidarians, tapeworms, flukes, roundworms and thorny‐headed worms (acanthocephalans). Although larval stages predominantly infect the internal viscera and may encyst within various host tissues, adult forms primarily colonize the host′s digestive tract [17, 19, 20].

Variations in the prevalence and intensity of internal parasites of fish are dependent on a variety of factors, including the species of host and feeding habits, the parasite species and life cycle, and the physical characteristics of the water body that the fish inhabits [21, 22]. The presence of intermediate hosts, such as snails and piscivorous birds, is also necessary for the parasites to spread to other hosts [23]. In addition to host and environmental factors, a lack of community awareness regarding fish health significantly exacerbates the distribution of fish parasite species and the severity of subsequent infections. For example, in Ethiopia, the risk of zoonotic transmission remains high due to frequent human infections with fish‐borne parasites, like *Diphyllobothrium*, often caused by eating raw or undercooked fish [12, 18, 24].

Internal parasites in fish production cause severe diseases, leading to high mortality rates and significant growth reduction or weight loss [12, 25]. Additionally, these infections diminish the visual appearance and quality of the fish, resulting in lower market value and high consumer rejection. This disturbs the balance between fish supply and demand [25, 26]. Furthermore, consuming fish carries some risk because it may harbor pathogenic zoonotic parasite species, particularly in areas where fish is consumed raw [27, 28].

For the fishing industry to remain sustainable and to harvest wholesome products of the highest caliber, it is imperative that all of the nation′s water bodies be routinely screened for pathogenic parasites [28]. Diagnostic methods for detecting internal parasitic helminthes in fish involve a combination of classical, molecular, and enhanced recovery techniques to ensure high diagnostic sensitivity. Although necropsy including macroscopic examination of viscera and musculature is fundamental, standard artificial digestion and incubation techniques are employed to break down fish tissue and release larvae, significantly increasing the likelihood of detecting helminth larvae. These methods employ artificial gastric juice, often comprising pepsin and hydrochloric acid, combined with controlled incubation and filtration to effectively isolate larval stages, such as *Anisakis* [29, 30].

Extensive research into fish parasitology has been well established across Europe, the Americas, and the Asia‐Pacific region including major contributions from Russia, China, and Australia yet such comparative studies in Africa have only recently begun to close this knowledge gap [26, 28, 31]. Also, there are still few studies which have not been well documented on the occurrence of internal parasitic helminthes of commercially viable fish species in Ethiopia′s natural waterbodies [10–12, 32, 33]. Although, Lake Chamo is a great contributor of fish source for the country, the internal parasitic helminthes that can affect commercially viable fish health and production are not studied broadly. Also, there is no previous report on the extent of internal parasitic helminthes infection in commercially viable fish species in Lake Chamo. The possible risk factors that enhance fish internal parasite infection and potential fish parasites that can easily be disseminated to these waterbodies are not sufficiently known. Furthermore, knowledge, awareness, and control practice of community on public health importance of fish internal parasite has not been studied in the current study area. Hence, this study was conducted with the aim to estimate the prevalence, identify associated determinants and assessment of the level of knowledge, awareness, and control practice of people towards internal parasitic helminthes of commercially viable fish species in Lake Chamo, Southern Ethiopia.

## 2. Materials and Methods

### 2.1. Description of the Study Area

**Figure 1:**
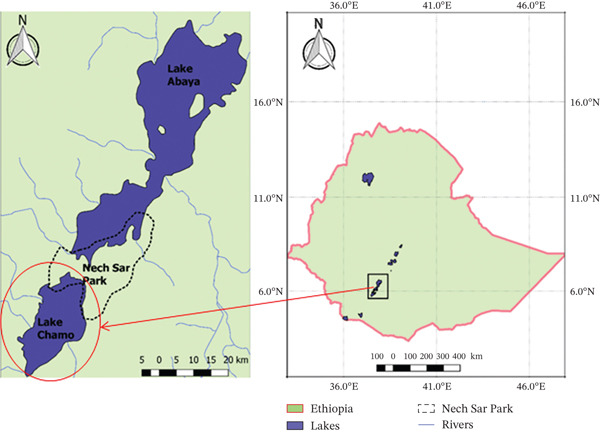
Map of Lake Chamo. *Source:* Adapted from Google Map 2025.

This study was carried out from October 2023 to December 2024 in Lake Chamo, Southern Ethiopia (Figure 1). Lake Chamo is a rift valley lake located south of Lake Abaya and the city of Arba Minch, nestled between the Guge and Amaro Mountains. Its northern end is within the Nechisar National Park. It is 32 km long and 13 km wide, with a surface area of 317 km^2^ and a maximum depth of 14 m with a catchment of about 18,757 km^2^ in size. Geographically, it is located at latitude of 5^°^42 ^′^–5°48 ^′^N and longitude of 36^°^30 ^′^–38^°^30  ^′^E 518 km south of Addis Abeba, the capital city of Ethiopia [8]. Lake Chamo has a highly diverse fish fauna and considered as the most diversified among the Ethiopian Rift Valley lakes, with over 20 species present. From these *L. niloticus*, *O. niloticus, Labeo horii, B. docmak, C. gareipinus, B. bynni, and H. vittatus* fish species are of great economic importance in fishing industry. Lake Chamo contributes the largest annual fish products of the rift valley lakes. It has a capacity of producing 4500 tons of fish per year [7, 12, 13, 34].

### 2.2. Study Population

The study population represents widely acceptable and commercially important fish species found in Lake Chamo including *O. niloticus, C. gariepinus, L. niloticus, B. docmak,* and *H. vittatus* [7, 12]. The respondents for the questionnaire survey, which includes fishermen and farmers living in and around the vicinity of Lake Chamo, were also considered as the study population.

### 2.3. Study Design

A cross‐sectional study was conducted from October 2023 to December 2024 to estimate prevalence and to assess potential determinants of internal parasitic helminthes of *O. niloticus, C. gariepinus, L. niloticus, B. docmak,* and *H. vittatus* fish species in Lake Chamo, Southern Ethiopia. Besides, a questionnaire survey was used to assess the awareness level of fishermen and farmers towards the public health importance of parasitic helminthes of fish in the study area.

### 2.4. Estimating the Prevalence of Internal Parasitic Helminthes of Fish

#### 2.4.1. Sampling Method and Sample Size Determination

Systematic random sampling technique was employed to select the *O. niloticus, C. gariepinus, L. niloticus*, and *B. docmak* as their larger population, whereas *H. vittatus* were conveniently trapped due to their limited numbers in Lake Chamo. According to de Blas et al. [35] in the case of aquatic animals, population size is considered “infinite”; so to estimate the prevalence, the required sample size (*n*) is calculated using the formula that depends on expected prevalence (*P*), accepted error (*E*), and 95% confidence level (1 − *α*, that determines the value of *Z* in a normal distribution), 5% desired absolute precision, and 50% expected prevalence due to the absence of information from previous studies in the study area.

*n*  =  ( *Z* ^2^ *a*/^2^ *P*(1 − *P*) / *E*) ^2^  =  (1.96^2^ *x*  0.5(1  −  0.5)/0.05) 2  =  369. Therefore, the required sample size was 369. To collect fish samples, the landing sites were visited three times in a week during late morning and/or afternoon as this was the time when the majority of fishermen arrived at the landing sites from the water. The study was performed on a total of 369; *O. niloticus* (258)*, C. gariepinus* (37)*, L. niloticus* (29)*, B. docmak* (32), and *H. vittatus* (13) fish species collected from local fisherman caught using different mesh sized gill nets.

#### 2.4.2. Study Methodology

##### 2.4.2.1. Fish Sample Collection, Transport and Handling.

Fish samples were bought following harvest and immediately transported within an icebox to the Veterinary Parasitology Laboratory, Arba Minch University for parasitological examination.

##### 2.4.2.2. Determination of Length, Weight, Age, and Sex of Fish.

The species, sex, body weight, total and standard lengths, and age of individual fish, as well as the season and predilection site of parasite was initially assessed and recorded [32,36–38]. Length and weight of fish were taken immediately after harvest using a ruler and measuring balance, respectively. The total and standard length of each fish which were measured by taking the length from the snout to the end of the caudal fin, and from the snout to the tip of the caudal peduncle, respectively, using a ruler [37]. Fish samples were weighed using weighing balance [39]. The total and standard body length of fishes was taken as small, medium, and large; body weight of fishes was categorized as light, medium, and heavy [32, 36]. The fishes were categorized into four groups according to their weight to determine their age groups classified as fingerlings, juveniles, young, and adult fish [36–38]. The sex of each fish was determined after dissection and noting the presence of testes or ovaries [40].

##### 2.4.2.3. Postmortem Examination of Fish for Parasitic Helminthes.

Immediately after fish were harvested, the postmortem examination was done using appropriate postmortem kits and standard evisceration technique [41]. Briefly, the whole body cavity was opened and examined for parasites in the intestinal tract and other sites including the muscles. Coelom was opened by making a ventral surface cut from the anus forward to an imaginary line at the posterior portion of the operculum, then cut out the entire side of the coelom by cutting a rectangle of skin from behind the operculum, anterior to the anus, and ventral to the backbone. Eventually, small and large intestines were cut out and placed in Petri dishes. The various organs, stomach, intestine, liver, heart, gall bladder, and gonads were removed and placed separately in Petri dishes containing 0.75% saline solution.

The skin around the eyes was cleaned with a sterile cotton wool and the eyes were gently pulled with forceps. The optic nerve was cut using scissors and the eyes were placed in a sterile Petri dish. The eye lens and vitreous were examined for the presence of *s*pecies of parasites under the microscope and presented as described by [42]. The brains were dissected longitudinally and the cranial cavity was washed away into a Petri dish using a water dropper and checked for parasites. Internal organs were checked for helminthes by naked eyes and microscopically. All the collected parasites were preserved in a plastic bag containing 4% formaldehyde solution and/or 70% ethanol [14].

##### 2.4.2.4. Identification of Parasitic Helminthes.

The parasites were identified under a light or stereomicroscope depending on the size of the parasite. The identification of parasites collected relied on (i) the comparison of distinctive body shapes and the morphological features of the collected specimen and those described in literature and (ii) the identification guideline as stated by standard keys in literature [39, 43]. The specimens were identified to genera level [32, 33, 36–38].

### 2.5. Identifying Determinants of Internal Parasitic Helminthes of Fish

Species, sex, body weight, age, total length, standard length of fish, and season were taken as potential determinants for the occurrence of fish internal helminthes infection in the study area [8, 12]. This information was recorded in the prepared data sheet for each fish sample.

### 2.6. Assessment of Public Awareness

Questionnaire was designed to collect information from fishermen and farmers to assess awareness level on public health importance of fish internal helminthes. The questionnaire consisted of questions related to sociodemographic facts, knowledge on fish parasites, raw fish consumption experience, and public health implications.

The sample size for the questionnaire survey was calculated based on Arsham [44] formula of *n* = 0.25/SE^2^, where *n* = sample size and SE = standard error. A standard error of 0.04 was taken to calculate the total respondent to be involved in the questionnaire survey, *n* = 0.25/(0.04)^2^ = 156. So, the sample size for the questionnaire survey was 156.

### 2.7. Data Management and Analysis

All data collected from the field survey and laboratory analysis were carefully arranged, coded, and recorded accordingly in Microsoft Excel spreadsheet. STATA Version 16.0 computer software was used for the statistical analysis at 95% confidence interval. Accordingly, descriptive statistics including frequencies and percentages were used to calculate the demographics of the farmers, their awareness, and the prevalence of fish parasites. Odds ratio was applied to see the strength of association between various risk factors with occurrence of fish internal parasitic helminthes. In all cases, the association was considered as significant when the *p* value at 95% confidence interval was less than 0.05 (*p* < 0.05).

## 3. Results

### 3.1. Overall Prevalence of Internal Parasitic Helminthes of Fish in Lake Chamo

In this study, a total of 369 fish samples were caught and examined for the presence of internal parasitic helminthes of fish in Lake Chamo, Southern Ethiopia. Out of the total sampled fish species, 250 were found infested with one or more internal parasitic helminthes, hence giving an overall prevalence of 67.8% in the study area (Table 1). Higher prevalence was observed in *O. niloticus* (80.6%), followed by *L. niloticus* (58.6%), *B. docmak* (46.9%), *C. gariepinus* (27%), and no parasites were identified in *H. vittatus* fish species.

**Table 1:** Overall prevalence of internal parasitic helminthes of fish in Lake Chamo.

Species of fish identified	Number of examined	Number of infected	Prevalence (%)
*O. niloticus*	258	208	80.6
*C. gariepinus*	37	10	27
*L. niloticus*	29	17	58.6
*B. docmak*	32	15	46.9
*H. vittatus*	13	0	0
Total	369	250	67.8

### 3.2. Prevalence of Internal Parasitic Helminthes of Fish in Relation to Organs Affected

The infection sites of internal parasitic helminthes of fish were recorded during the study period of sample examination. Among the organs observed, pericardial cavity, intestine, eye, gill, abdominal cavity, mesentery, liver, muscle, and ovary were found to be infected. Pericardial cavity was found as dominantly affected organ (62.4%) followed by intestine (56%) (Table 2)

**Table 2:** Prevalence of internal parasitic helminthes of fish in relation to organs affected.

Organs affected	Fish species				Prevalence from overall infection (250)
	O. *niloticus*	*C. gariepinus*	*L. nilotius*	*B. docmak*	
Pericardial cavity	144	5	5	2	156 (62.4%)
Intestine	132	8	0	0	140 (56%)
Eye	19	0	0	0	19 (7.6%)
Gill	16	0	0	0	16 (6.4%)
Abdominal cavity	0	0	0	13	13 (5.2%)
Mesentery	0	0	12	0	12 (4.8%)
Liver	6	0	3	0	9 (3.6%)
Muscle	2	3	0	0	5 (2%)
Ovary	2	0	0	0	2 (0.8%)

### 3.3. Identified Genera of Fish Internal Parasitic Helminthes

**Figure 2:**
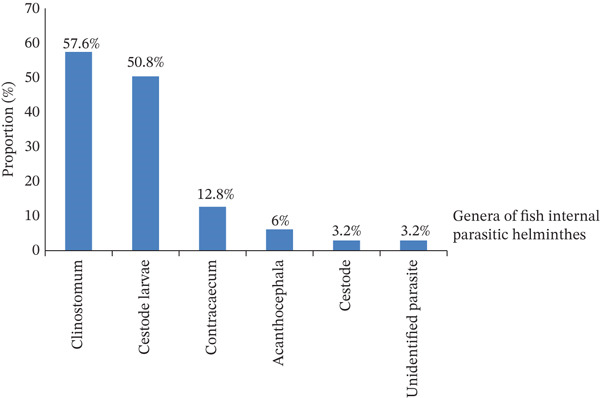
Identified genera of fish internal parasitic helminthes.

Microscopic examinations of samples collected from fish internal organs revealed different genera of internal helminthic parasites of fish. Generally, six genera of internal parasitic helminthes of fish were identified from Lake Chamo during the study period. These were *Clinostomum* (57.6%), cestode larvae (50.8%), *Contracaecum* (12.8%), Acanthocephala (6%), cestode (3.2%), and unidentified genera of fish parasite (3.2%) in descending order as shown in Figure 2. Also, out of 250 infested fish samples, 168 (67.2%) had helminth infection harboring at least one helminth parasite and 99 (31.4%) had multiple infections (Table 3).

**Table 3:** Type of parasite infection demonstrated in fish in study area.

Type of infection	Frequency	Percentage (%)
Single	168	67.2
Mixed	82	32.8
Total	250	100

### 3.4. Prevalence of Helminthes Parasites in Relation to Each Fish Species

The frequency of occurrence and prevalence of helminth parasites identified in relation to each fish species in this study is summarized on Table 4. This result revealed that *Clinostomum* and Cestode larvae parasites were identified only in *O. niloticus* with a prevalence of 55.8% and 49.2%, respectively. The prevalence of *Contracaecum* parasites was higher in *L. niloticus* (58.6%) than in *C. gariepinus* (8.1%) and *O. niloticus* (4.7%) fish species. Acanthocephala parasites were only recovered from *B. docmak* fish with a prevalence of 46.9%. Also, adult cestode and encysted trematode metacercaria parasites were only identified from *C. gariepinus* fish species with a prevalence of 21.6% and 8.1%, respectively.

**Table 4:** Genera of internal helminthes identified in each fish species.

Parasitic genera identified	Fish specious			
	*O. niloticus*	*C. gariepinus*	*L. niloticus*	*B. docmak*
*Clinostomum*	144 (55.8%)	0	0	0
Cestode larvae	127 (49.2%)	0	0	0
*Contracaecum*	12 (4.7%)	3 (8.1%)	17 (58.6%)	0
Acanthocephala	0	0	0	15 (46.9%)
Adult cestode	0	8 (21.6%)	0	0
Encysted trematode metacercaria	0	3 (8.1%)	0	0
Unidentified parasite	3 (1.2%)	0	2 (6.9%)	0

### 3.5. Determinants Associated With Occurrence of Internal Parasitic Helminthes of Fish

Season, species, sex, body weight, age, total length, and standard length of fish were considered and taken as potential risk factors for the occurrence of internal parasitic helminthes of fish in the study area by univariable logistic regression analysis (Table 5). The variation in the prevalence of internal parasitic helminthes of fish was statistically significant between species, sexes, and standard body length. However, the variation between age, body weight, total body length, and season was found to be statistically insignificant. Also, this result showed that the odds of infection were statistically significant, with more likelihood in *O. niloticus* (OR = 2.9, CI = 1.32–6.54), female (OR = 1.6, CI = 1.03–2.49), and in large standard length fish (OR = 2.2, CI = 1.15–4.16) compared with their respective categories.

**Table 5:** Univariable logistic regression analysis of determinants associated with occurrence of internal parasitic helminthes of fish.

Variables	Category	Number of examined	Number of infected	Prevalence (%)	OR	CI	*p* value
Fish species	*L. niloticus*	29	17	58.6	Ref	—	—
	*O. niloticus*	258	208	80.6	2.9	1.32–6.54	0.008
	*C. gariepinus*	37	10	27	0.3	0.09–0.74	0.011
	*B. docmak*	32	15	46.9	0.6	0.23–1.72	0.360
	*H. vittatus*	13	0	0	1	—	—
Sex	Male	154	95	61.7	Ref	—	—
	Female	215	155	72.1	1.6	1.03–2.49	0.036

Age	Fingerling	74	49	66.2	Ref	—	—
	Juvenile	104	66	63.5	0.9	0.47–1.66	0.705
	Young	83	58	69.9	1.2	0.60–2.32	0.623
	Adult	108	77	71.3	1.3	0.67–2.40	0.466

Body weight	Light	187	125	66.8	Ref	—	—
	Medium	100	65	65	0.9	0.55–1.53	0.753
	Heavy	82	60	73.2	1.4	0.76–2.41	0.304

Total length	Small	83	55	66.3	Ref	—	—
	Medium	186	116	62.4	0.8	0.49–1.45	0.540
	Large	100	79	79	1.9	0.99–3.71	0.054

Standard length	Small	81	50	61.7	Ref	—	—
	Medium	184	119	64.7	1.1	0.66–1.95	0.646
	Large	104	81	77.9	2.2	1.15–4.16	0.018

Season	Wet	197	137	69.5	Ref	—	—
	Dry	172	113	65.7	0.8	0.54–1.29	0.431

Abbreviations: Ref, reference; OR, odds ratio; CI, confidence interval.

### 3.6. Questionnaire Survey Analysis

#### 3.6.1. Sociodemographic Characteristics of Respondents

A survey of 156 fishermen and farmers in the study area was conducted to assess their awareness towards public health importance of parasitic helminthes of fish (Table 6). The majority of key participants in the study were male, married, fishermen, and in the adult age group. Also, among the fishermen, 59.6% had greater than 8 years fishing experience, and the highest percentage of respondents were educated.

**Table 6:** Sociodemographic characteristics of the respondents (*n* = 156).

Variables	Category	Number of respondent	Percentage (%)
Sex	Female	16	10.
	Male	140	89.7

Age	Young	3	1.9
	Early adulthood	62	39.7
	Adult	65	41.7
	Old	26	16.7

Marital status	Single	35	22.4
	Married	121	77.6
Educational status	Educated	144	92.3
	Illiterate	12	7.7
Occupation	Fisherman	114	73.1
	Farmers	42	26.9

Fishing work experience	At least 1 year	13	11.8
	At least 2 years	28	24.2
	4–7 years	5	4.4
	> 8 years	68	59.6

#### 3.6.2. Respondents Awareness Level on Public Health Importance of Internal Parasitic Helminthes of Fish in Study Area

Table 7 reveals respondents′ knowledge and awareness level towards the public health importance of internal parasitic helminthes of fish in the study area. Out of 156 surveyed respondents, 95.5% knew the presence of fish parasites and 89.9% of respondents had knowledge about fish‐borne zoonosis. Among the respondents that have previous knowledge of the presence of fish parasites, 73.8% encountered the internal helminthes in the fish body. Also, 84.5% of respondents stated that among fish species, *Nile tilapia* were mostly affected by internal fish parasites than other commercially viable fish species in Lake Chamo. With regard to eating raw fish, 90.4% of the respondents were consuming raw fish, and *Nile tilapia* followed by *Nile perch* are preferable fish species to be eaten raw by the majority of respondents in the study area. From the respondents that have been consuming raw fish, 22.7% stated that they have experienced gastrointestinal problems after eating raw fish. Also, among respondents who experienced gastrointestinal problems, 81.3% believed that the disease was from eating raw fish. Furthermore, 64.2% of respondents think of drinking fermented alcohol and 35.8% using lemon as prophylactic treatment after consumption of raw fish.

**Table 7:** Respondents′ knowledge and awareness level towards public health importance of internal parasitic helminthes of fish in the study area.

Variables	Category	Number of respondent	Response (%)
Knowledge about fish parasites	Yes	149	95.5
	No	7	4.5
Encounter a parasitic infection in a fish	Encountered	110	73.8
	Did not encounter	39	26.2

Fish species most commonly affected by parasites	Koda (*Nile tilapia*)	93	84.5
	Ambaza (*African catfish*)	9	8.2
	Nech asa (*Nile perch*)	4	3.6
	Kerkero (*Bargus docmak*)	4	3.6

Practices done after harvesting fish infested by internal parasites	Removed the parasite and sell to the consumer	49	44.5
	Gave the fish to birds	40	36.4
	Remove the parasite and used for home consumption	13	11.8
	Throw the fish to water	6	5.5
	Simply sold infected fish to the consumer	2	1.8
Consuming practices of raw fish	Yes	141	90.4
	No	15	9.6
Raw fish species consumed	Koda (*Nile tilapia*)	141	100
	Nech asa (*Nile perch*)	70	44.9
Knowledge about fish‐borne zoonosis	Yes	134	89.9
	No	15	11.1
Experience of disease condition after eating raw fish	Yes	32	22.7
	No	109	77.3

Disease signs experienced after consuming raw fish	Colic	13	40.6
	Diarrhea	7	21.9
	Vomiting	4	12.5
	Both colic and diarrhea	8	25

Believing experienced disease condition was due to eating of raw fish	Yes	26	81.3
	No	6	18.7

Control method used for fish parasite zoonosis	Drinking alcohol	86	64.2
	Drinking lemon	48	35.8
	Other methods	0	0
Fish eviscerating places by fisherman	At shore of lake	114	100
	Other places	0	0

Sanitation methods practiced by fisherman	Cleaning working environment and equipment	97	85
	Burning waste material	73	64.2
	Disinfection	24	20.8

## 4. Discussion

Fish internal parasitic helminthes can significantly impact fish health, production, and also some have public health importance. Knowing and comprehending the nature of prevalence and determinants of internal parasitic helminthes of commercially viable fish species is essential for developing effective disease prevention strategies [5, 7, 12, 31]. Because the current study relied only on visual inspection rather than incubation or digestion methods and other molecular techniques, our findings likely underestimate the true parasite prevalence. Future research should use more sensitive diagnostic techniques.

In the present study, a total of 369 different commercially viable fish species were examined for the presence of internal parasitic helminthes, out of which 250 (67.8%) were found infected by internal parasitic helminthes. This finding was in agreement with previous reports done by [10, 45] who indicated an overall prevalence of 66.3% and 69.25%, respectively, in the Koka reservoir and Zeway Lake of Ethiopia. Also, relatively similar to the current study, Kawe *et al.* [46] reported a 67.5% prevalence of gastrointestinal helminthic parasites from *C. gariepinus* in Abuja, Nigeria. On the contrary, the current finding is much higher than the findings in previous studies carried out in different water bodies of Ethiopia: 37.6% by [15], 42.3% by [33], 47.8% by [5], and 57.3% by [11]. Also, these findings were higher than the study done by [1] from Upper Egypt who reported an overall prevalence of 41%.

However, the present finding is lower than Abiyu et al. [11] and Hussein *et al.* [23] who reported prevalence of 80% and 76.04%, respectively, in Lake Koftuin and in Lake Hawassa of Ethiopia. These variations in the prevalence of gastrointestinal helminthic parasites of fish across different parts of Ethiopia and between countries might be due to the differences in the distribution of parasites from one area to another; and a number of factors which include differences in physical and chemical conditions of the water, season, climatic conditions of the areas, and host–parasite relationship [5, 7, 47]. Fish caught by fishermen were eviscerated along the shore and washed into the lake and eaten by piscivorous birds, resulting in the recontamination of the lake, which increases the parasite burden [24]. The current high overall prevalence of internal parasitic helminthes of fish also might be due to the maturation of parasites in a large number of piscivorous birds around Lake Chamo; this allows parasites to reproduce more and infect a large number of fish hosts [47].

Furthermore, in the present study, we report a high overall prevalence (67.8%) of helminthic parasite infection in commercially viable fish species from Lake Chamo, which are locally consumed by surrounding communities and also reach key urban markets, including Addis Ababa, Ethiopia. Although much of this fish catch supports local consumption patterns with limited formal trade, it is important to contextualize our findings within broader fish trade and public health frameworks. Commercial fish trade both domestic and international can act as a conduit for the spread of pathogens and parasites, thereby influencing disease dynamics beyond the point of capture. Recent analyses indicate that global fishery trade may facilitate the movement of pathogens carried by aquatic products, highlighting implications for biosecurity and public health monitoring in regions connected by trade networks [48]. Studies on imported fish products have also documented the presence of zoonotic helminthes, such as anisakid nematodes and other parasite taxa, underscoring the potential for invasive parasite introduction and human exposure through consumption of contaminated fish. Although Lake Chamo fish are not widely exported internationally, even domestic distribution into urban centers increases the risk of exposing a larger consumer base to fish‐borne parasites [49, 50].

Pericardial cavity was found as the dominantly affected organ of fish (62.4%) followed by intestine (56%). This result agrees with the finding of [51], who revealed pericardial cavity as the most affected site by endoparasites in Nile tilapia and small *Barbus* fish species. This result disagrees with the findings of [52] who report gut harbors 86% of the parasites in *O. niloticus* and *Cyprinus carpio* in Lake Hashenge. This is due to high prevalence of clinostomum trematode parasite which prefers the pericardial cavity as predilection site. The second dominantly affected site was the intestine; this could be associated with the fact that the intestine acts as a food storage cavity which could probably result in the release of the ova or cysts of parasites in the food the fish swallow [53].

From different identified internal parasites, most fish suffered from a single type of parasite infection. Similarly, Yesuf et al. [33] found that single infection was the predominant case in selected lakes of South Wollo zone. In contrast, the report of [5, 54] found multiple infestations of parasites at Lake Lugo and in South West Showa zone selected fish farms, respectively. And it is the fact that the environment supports several parasites species thereby exposing the host to simultaneous infection with many of them. The presence of one parasite and its activity within the host weakens the resistance, which makes concurrent infection feasible [33].

The proportion of *Clinostomum* was highest (57.6%) followed by cestode larvae (50.8%). It is higher than the finding of [5] who revealed *Contracaecum* (42.6%) were the most frequently found parasite followed by *Clinostomum* (38.6%) in Lake Lugo of Northeast Ethiopia. But it is lower than the result (69.25%) of [45] in Lake Zeway, Ethiopia. The proportion of *Contracaecum* was 12.8%. This is line with the finding of [25] who found 11% in the Lake Kyoga, Uganda. But lower than the study of [51] who found 49.5% in Lake Tana, Ethiopia. The proportion of *Acanthocephalues* was 6%, which is lower than result (11.2%) of [15] in Lake Hashenge, Tigray, Ethiopia. The proportion of cestode was 3.2%, which is similar with the finding of [55] in Sohag governorate of Egypt. It is less than the finding of [56] who reported 35.4% in Qena governorate. These differences were associated with geographical variation, environmental factors and different level of intermediate hosts [7, 53].

Based on genera of internal helminthes identified in each fish species, *Clinostomum* was identified only from Nile tilapia with a prevalence of 55.8%. This result was similar to the previous report of [57] as 56.20% in Lake Zewai. However, it was higher than 23.3% recorded by [33] in selected lakes of South Wollo zone. Also, it is higher than the findings of [58] who reported *Clinostomum* occurred only in *O. niloticus* with a very low prevalence of 0.7% compared with the Winam Gulf of Lake Victoria of Kenya. It is lower than the study conducted by [45] with a prevalence of 69.25% in Lake Zeway, Ethiopia. This might be due to the geographical area, disposal of fish offal into the lake tending to harbor more internal helminthes than those from less polluted environments [53].

The prevalence of cestodes in *C. gariepinus* was 21.6% in this study lower than the reports of [10, 23, 56], respectively, with the prevalence of 50.7%, 35.86%, and 35.4% in different lakes of Ethiopia. Acanthocephala (46.9%) recovered from *B. docmak* was higher than the works of [10], who reported prevalence of 2.73% in Koka Reservoir of Ethiopia. Also, it is higher than findings of [38] which were 15.8% in *Bagrid* fishes in Anambra river Basin of Nigeria. These differences are associated with variation in geographical and environmental factors, time interval between the previous studies and level of intermediate hosts [7, 10, 53, 56].

In Nile tilapia, numerous cysts in the intestinal wall containing cestode larvae were observed with the prevalence of 49.2% which is similar to the report done by [59] in Kenya, Uganda, and Ethiopia. Macroscopic encysted trematode metacercaria (8.1%) was found in the *C. gariepinus* fish; this finding is similar to the finding of [60] in Lake Manzala and Nile River canals in the Giza governorate, Egypt. However, this finding was lower than [1], who reported a prevalence of 37% in freshwater fish from upper Egypt. The difference is directly related to variations in geographical and environmental factors [53].

The current study identified species of fish as a significant risk factor. The highest proportion was recorded in *O. niloticus* followed by *L. niloticus, B. docmak*, and *C. gariepinus,* respectively. This finding is line with the finding of Bedasso [61] who reported highest proportion in *O. niloticus* followed by *L. intermius* in Lake Belbela, Ehiopia. This result disagrees with the study of Hailu et al. [62] who found *C. gariepinus* had the high proportion of parasitic infestation, followed by *O. niloticus* in Lake Ziway, and Tesfaye et al. [32] who revealed higher proportion in *C. gariepinus* followed by *O. niloticus* in Lake Hawassa, Ethiopia. This variation in proportion of parasites in the different fish species might be attributed to the different habitat use and behavior of diet.

Furthermore, Mitiku et al. [24] suggested that *O. niloticus* forms and defends territories along the shores. This territorial behavior increases the proximity to and maintains continuous exposure to helminthes [63]. *O. niloticus* feeds mainly on phytoplankton and macrophytes although zooplankton and benthic organisms also contribute to the diet [64]. Because zooplankton and benthic organisms act as intermediate hosts for several helminthes, their intake exposes the fish to infections. The *C. gariepinus* fish species prefers marginal weedy and muddy waters, and *L. niloticus* and *B. docmak* feed on a wide range of food items including detritus, zooplankton, insects, and fish. All of these can be act as intermediate hosts for several helminthes; however, it might be limit the intake of the parasites [65].

In this study, no infection was recorded in *H. vittatus*. This result is line with [66] who reported absence of recorded parasite infection in *Barbus* species in Gilgel Gibe River and three selected ponds in and around Jimma Town, South West Ethiopia. The community near to Lake Chamo has been used only old and adult *H. vittatus* fish species. So, fisherman has been catching only old and adult *H. vittatus*. In addition to these, the population of old and adult *H. vittatus* was limited in Lake Chamo [12]. Due to these, the collected sample size of *H. vittatus* was very small. These reasons might be contributed to make difficult to get positive *H. vittatus* fish species.

The current study also identified sex of fish as a significant risk factor. The odds of getting infection in female fish was 1.6 times higher when compared with female fish (OR = 1.6; CI = 1.03–2.49). This corresponds to the findings of [5] in Lake Lugo, Northeast Ethiopia; and [15] in Lake Hashenge, Ethiopia who stated that female fish were generally more liable than males to infestations with various genera of fish internal helminthes parasites. This might be due to the fact that female can exposed for various stressing factor acquiring from searching of feed and shelter for preparing to be ready for oviposition, this may decrease the immune system and leads to parasitic infestation [3, 7]. However, Akinsanya et al. [67] in Lake Lagoon of Nigeria, and Allumma and Idowu [37] in Baga side of Lake Chad found that male fish had higher proportion of internal parasite infestation than females which contradicts with this finding. It was justified by [33] to be due to the exhaustion of male′s immunity by frequent insemination of females when the fish stock is available.

The current study showed that one of the determinants for internal parasitic helminthes of fish in the study area was the standard length. Higher prevalence of internal parasitic helminthes was observed in fish with larger standard length (77.9%) than fish with medium (64.7%) and small standard length (61.7%). That means the likelihood of contracting internal parasitic helminthes increases with standard length. For instance, the likelihood of getting parasitic helminthes in fish with larger standard length was 2.2 times greater than fish with small standard length (OR = 2.2; CI = 1.15–4.16). It concurs with the previous study carried out by [23, 32, 37, 68] in different lakes of Ethiopia and Nigeria. Pal et al. [5] explained that prevalence of parasite infection increased with increasing length and size of the fish host as it increases their chance of acquiring the parasite infection with time and increased size as well. This is also due to longer fish provides greater surface for infection than smaller ones [3, 7, 17, 18].

Out of 156 surveyed respondents, 95.5% knew the presence of fish parasites and 89.9% of respondents had knowledge about fish‐borne zoonosis. This result is similar to the finding of [69] who reported the majority (72.1%) of respondents knew that diseases could be transmitted from fish to humans. Among the respondents that have previous knowledge of the presence of fish parasites, 73.8% encountered the internal helminthes in the fish body. Also, 45.5% of the respondents who encountered the internal parasitic helminthes in the fish body agreed that the infection of parasites can damage fish health as they encountered weak and thin fishes with hemorrhagic appearance that were suffering from internal parasites at different times in Lake Chamo fish harvests. This result was similar to the findings of [69], who reported that 41.6% of people living near Lake Hashenge agreed on the presence of fish parasites that damage fish health. But it was lower than the reports of [62] who stated 100% of the respondents agreed on the presence of fish parasites which continue to be a nuisance in the processing and marketing of Lake Ziway fish harvests. These findings suggest that awareness regarding fish internal parasitic helminthes as a zoonotic disease is relatively widespread in the study area.

Major of respondents (84.5%) stated that among fish species, Nile tilapia were most affected by internal fish parasites than other commercially important fish species in Lake Chamo. This result agrees with the results of [51] at Lake Tana of Ethiopia. Oppositely, [70] reported a higher prevalence of endoparasite infection in *C. gariepinus* than *O. niloticus* at Ziway of Ethiopia. This variation in prevalence of parasites in the different fish species might be attributed to the different habitat use and behavior of feeding [63]. Among the respondents who have encountered fish with internal helminthes, 44.5% simply removed the parasite and sold it to the consumer, and 36.4% practiced giving away the infected fish to birds. These findings suggest that the community near Lake Chamo has poor awareness of the way parasites spread. This result is similar to the works of [12] in the current study area.

With regard to eating raw fish, 90.4% of the respondents were consuming raw fish. This finding is in close agreement with the works of [12] at the same Lake and [62] that reported people′s tradition of eating raw fish and poorly prepared fish in Lake Ziway, Ethiopia. It is therefore likely that, with such a high abundance of parasites in Lake Chamo fish and the raw fish consumption habits of the people in the study area, there is a failure in the practical application of preventive measures and some of the zoonotic fish parasitic diseases have gone unnoticed. Also, the majority of respondents stated that Nile tilapia and Nile perch fish species are preferable fish species to be eaten raw. This result was in agreement with the findings of [12] at the same lake. Fish species preference for raw fish has also been reported in Vietnam [71]. This might be due to their culturally acceptable fish species for human consumption.

Even if they have awareness of fish parasites that can affect human health, 64.2% of respondents think of drinking fermented alcohol, and 35.8% use lemon as prophylactic treatment after consumption of raw fish. This result is similar to the study reported by [12] at the same lake. This might suggest misunderstanding among the community about control practices. Among respondents that have been consuming raw fish, some (22.7%) stated that they have experienced gastrointestinal problems after eating raw fish. Among respondents experiencing gastrointestinal problems, 81.3% believed that the disease was from eating raw fish and the rest 18.7% of respondents did not believe it was from eating raw fish. As they stated, the prevalent gastrointestinal problem signs among these respondents are colic, diarrhea, and vomiting. This might agree with the report of [72] who stated most individuals infected by fish internal parasites may have weight loss, diarrhea, anemia, colic, and vitamin B12 deficiency.

## 5. Conclusions

The present study identified various types of internal parasitic helminthes mainly, *Clinostomum*, Cestode larvae, *Contracaecum*, Acanthocephala, and cestodes in commercially viable fish species harvested from Lake Chamo of Southern Ethiopia with the overall prevalence of 67.8%. Also, unidentified parasitic helminthes were found in this survey. Species, sex, and standard length of fish were found to be potential determinants for the occurrence of fish helminthes in the study area. The finding of *Clinostomum* and *Contracaecum* species is indicative of the existing risks of public health. However, most of the people living near Lake Chamo have been eating raw fish, and there was a critical knowledge gap in the practical application of preventive measures among people. Therefore, for effective management and control of internal parasitic helminthes of commercially viable fish species in the current study area, an integrated approach should be promoted that takes into account the relationship between fish fauna, humans, and Lake Environment in the context of “One Health approach principles.” Further investigations including all fish parasites by considering additional risk factors and also molecular studies that address the biology and zoonotic potential of internal parasitic helminthes of fish species should be carried out in Lake Chamo of Southern Ethiopia.

## Author Contributions

All the stated authors of this manuscript have made a significant contribution to conception, study design, execution, and acquisition of data, analysis and interpretation. Again, all the authors took part in drafting, revising, or critically reviewing the manuscript.

## Funding

No funding was received for this manuscript.

## Disclosure

All authors took part in giving final approval of the version to be submitted to the agreed journal. Moreover, they all agreed to be accountable for all aspects of the work.

## Ethics Statement

The research was carried out with high regard for animal welfare and ethical approval was obtained from the Animal Research Ethics review committee of Arba Minch University (Ref. No. AMU/AREC/20/2018). Before collecting fish samples, the study objectives were explained to the human participants (fishermen and farmers) living in and around Lake Chamo vicinity. Verbal consent was then obtained from them to ensure their willingness to respond to the questions in the data collection sheet. Verbal informed consent was reviewed and approved by the Animal Research Ethics Review Committee of Arba Minch University. All best animal health practices, proper guidelines, and regulations were applied.

## Conflicts of Interest

The authors declare no conflicts of interest.

## Data Availability

The data that support the findings of this study are available from the corresponding author upon reasonable request.
